# Host cell response and distinct gene expression profiles at different stages of *Chlamydia trachomatis* infection reveals stage-specific biomarkers of infection

**DOI:** 10.1186/s12866-020-02061-6

**Published:** 2021-01-04

**Authors:** Emmanuel Enoch Dzakah, Liping Huang, Yaohua Xue, Shuai Wei, Xiaolin Wang, Hongliang Chen, Jingwei Shui, Foster Kyei, Farooq Rashid, Heping Zheng, Bing Yang, Shixing Tang

**Affiliations:** 1 Dermatology Hospital of Southern Medical University, Guangzhou, China; 2 Department of Molecular Biology and Biotechnology, School of Biological Sciences, College of Agriculture and Natural Sciences, University of Cape Coast, Cape Coast, Ghana; 3 Guangdong Provincial Key Laboratory of Tropical Disease Research, School of Public Health, Southern Medical University, Guangzhou, China; 4 Hefei National Laboratory for Physical Sciences at Microscale, the CAS Key Laboratory of Innate Immunity and Chronic Disease, School of Life Sciences, University of Science and Technology of China, Hefei, China; 5 The First People’s Hospital of Chenzhou, University of South China, Chenzhou, Hunan China

**Keywords:** *Chlamydia trachomatis*, Differentially expressed genes, Biomarkers, Signaling pathways, Replication

## Abstract

**Background:**

*Chlamydia trachomatis* is the most common sexually transmitted infection and the bacterial agent of trachoma globally. *C. trachomatis* undergoes a biphasic developmental cycle involving an infectious elementary body and a replicative reticulate body. Little is currently known about the gene expression dynamics of host cell mRNAs, lncRNAs, and miRNAs at different stages of *C. trachomatis* development.

**Results:**

Here, we performed RNA-seq and miR-seq on HeLa cells infected with *C. trachomatis* serovar E at 20 h post-infection (hpi) and 44 hpi with or without IFN-γ treatment. Our study identified and validated differentially expressed host cell mRNAs, lncRNAs, and miRNAs during infection. Host cells at 20 hpi showed the most differential upregulation of both coding and non-coding genes while at 44 hpi in the presence of IFN-γ resulted in a dramatic downregulation of a large proportion of host genes. Using RT-qPCR, we validated the top 5 upregulated mRNAs and miRNAs, which are specific for different stages of *C. trachomatis* development. One of the commonly expressed miRNAs at all three stages of *C. trachomatis* development, miR-193b-5p, showed significant expression in clinical serum samples of *C. trachomatis*-infected patients as compared to sera from healthy controls and HIV-1-infected patients. Furthermore, we observed significant upregulation of antigen processing and presentation, and T helper cell differentiation pathways at 20 hpi whereas T cell receptor, mTOR, and Rap1 pathways were modulated at 44 hpi. Treatment with IFN-γ at 44 hpi showed the upregulation of cytokine-cytokine receptor interaction, FoxO signaling, and Ras signaling pathways.

**Conclusions:**

Our study documented transcriptional manipulation of the host cell genomes and the upregulation of stage-specific signaling pathways necessary for the survival of the pathogen and could serve as potential biomarkers in the diagnosis and management of the disease.

## Background

*Chlamydia trachomatis* (*C. trachomatis*) is an obligate intracellular bacterial agent that is considered as one of the most widespread sexually transmitted infections (STIs). *C. trachomatis* is usually transmitted through risk behaviors similar to human immunodeficiency virus type one (HIV-1) infection in humans [1–4]. Annually, more than 127 million cases of *C. trachomatis* infection occur worldwide [1]. *C. trachomatis* generally undergoes a biphasic developmental cycle involving an infectious elementary body (EB) and a replicative form called the reticulate body (RB) [5, 6]. When the EBs get into contact with the host cell, they are internalized to form membrane-bound bodies (inclusion bodies). The EBs then differentiate into RBs that undergo binary fission forming large vacuoles in the host cell. Under unfavorable conditions, such as in the presence of IFN-ɣ, the pathogen differentiates into a quiescent noninfectious aberrant structure termed the “persistent” form or aberrant reticulate bodies (ARB) [7–9].

The developmental cycle of *C. trachomatis* is largely dependent on the host cell since the pathogen derives its nutrients from the host cell leading to changes in protein synthesis and host cell signaling pathways [6, 10]. Thus, the effective manipulation of the host cell’s metabolic and immune systems is crucial to the intracellular survival of *C. trachomatis* [11, 12]. The host cell genome responds to these changes by altering the expression of essential genes and cytokines to maintain proper function and survival of cells, as well as playing important roles in the clearance of the pathogen [13–15]. Inhibition of protein synthesis leads to the reprogramming of the host cells during *C. trachomatis* infection [15, 16]. Dramatic changes in the host cell genomes may result in a different outcome of antibiotic treatment of *C. trachomatis* and the existence of the persistent forms of the bacteria. Additionally, it was suggested that both chlamydial and host genes may synergistically function during host cell invasion [6]. Previous studies have mainly focused on Chlamydial transcriptomes over the developmental cycle in different chlamydial species [17–19]. Epithelial cell transcriptome in response to plasmid-bearing/plasmid-less *C. trachomatis* was previously characterized by microarray [20]. A recent report also indicated that *C. trachomatis*-infected host cells exhibit changes in protein synthesis and other host cell signaling pathways [21, 22].

There is currently a limited understanding of the changes in the expression levels of both protein-coding and non-coding genes in host cells at the different stages of *C. trachomatis* infection. The application of next-generation sequencing (NGS) techniques may provide a more accurate host cell genome-wide characterization of both coding and non-coding genes at various stages of infection. Here, we infected Hela cells with *C. trachomatis* and collected the inclusion body-containing host cells at 20 and 44 h post-infection (hpi), and also in the presence of interferon-gamma (IFN-γ) at 44 hpi. Using RNA-seq and miR-seq, the dysregulated host cell mRNAs, lncRNAs, and miRNAs, as well as the activated signaling pathways were documented in *C. trachomatis*-infected host cells at 20 hpi and 44 hpi with or without IFN-γ treatment. Analysis of serum miRNAs in healthy, *C. trachomatis*-infected, and HIV-1-infected patients showed a significant expression of miR-193b-5p only in *C. trachomatis*-infected patients. These findings highlight the gene expression dynamics and the dysregulation of *C. trachomatis* stage-specific signaling pathways in host cells during *C. trachomatis* infection and provide insights into potential biomarkers for improved clinical diagnosis and management of *C. trachomatis* infection.

## Results

### Host gene expression patterns during *C. trachomatis* infection

**Fig. 1:**
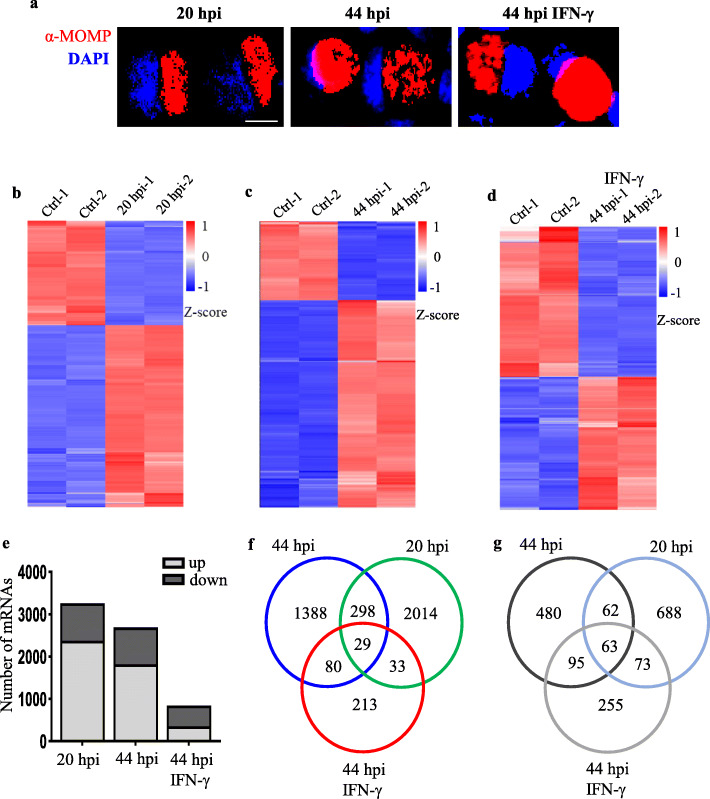
Genome-wide analysis of mRNAs at different stages of *C. trachomatis* infection. **a** Microscopic images of *C. trachomatis* infected cells at 20 hpi, 44 hpi, and IFN-γ treatment at 44 hpi. Red: anti-MOMP antibody, blue: DAPI. Fluorescence microscopy was carried out using Leica DM IL LED microscope (Wetzlar, Germany) and processed with Image J (https://imagej.nih.gov/ij/). Scale bar indicates 10 μm. **b-d** Heatmap plots showing differentially expressed host cell mRNAs at 20 hpi, 44 hpi, and 44 hpi with IFN-γ treatment as compared to their respective negative controls. Cutoff value > 1 reads and *P*-value < 0.05. Heatmaps were created with R version 3.6.3 (https://www.r-project.org/). **e** The number of host cell DEGs at 20 hpi, 44 hpi, and 44 hpi with IFN-γ treatment. **f** Venn diagram of upregulated mRNAs at 20 hpi, 44 hpi, and 44 hpi with IFN-γ treatment. **g** Venn diagram downregulated mRNAs in *C. trachomatis*-infected host cells. The differentially expressed genes are defined as those with fold change ≥2 (upregulated) or ≤ 0.5 (downregulated) between the groups and their respective controls with an adjusted P-value < 0.05. Data are the average expression from two independent experiments

To understand how *C. trachomatis*-infected host cells respond to infection at the various stages of *C. trachomatis* development, we infected HeLa cells with *C. trachomatis* serovar E. Cells were harvested at 20 hpi, 44 hpi without IFN-γ treatment or 44 hpi with IFN-γ treatment, mimicking the RB, EB, and ARB stages of *C. trachomatis* development, respectively (Fig. 1a). Total RNA extraction was followed by RNA-seq and miR-seq. Gene expression dynamics revealed significant dysregulation of host cell mRNAs, lncRNAs, and miRNAs at 20 hpi, 44 hpi, and at 44 hpi with IFN-γ treatment as compared to the corresponding uninfected controls. A total of 3260 differentially expressed genes (DEGs) comprising of 2374 (72.8%) upregulated and 886 (27.2%) downregulated genes were identified in *C. trachomatis*-infected cells at 20 hpi (Fig. 1b, e-g; Supplementary Fig. 1a). A total of 2663 DEGs including 1795 (67.4%) upregulated and 868 (32.6%) downregulated mRNAs were identified from host cells infected at 44 hpi compared with uninfected controls (Fig. 1c, e-g; Supplementary Fig. 1b). At 44 hpi with IFN-γ treatment, we observed 841 DEGs with 355 (42.2%) upregulated and 486 (57.8%) downregulated mRNAs (Fig. 1d-e; Supplementary Fig. 1c). Interestingly, 298 upregulated mRNAs were shared between host cells after 20 hpi and 44 hpi as compared to 80 mRNAs at 44 hpi with or without IFN-γ treatment, and only 33 mRNAs shared by the host cells at 20 hpi and IFN-γ treatment at 44 hpi. A total of 2014, 1388, and 213 mRNAs were specifically upregulated at 20 hpi, 44 hpi without IFN-γ, and 44 hpi with IFN-γ treatment, respectively (Fig. 1f). Of note, only 29 upregulated and 63 downregulated mRNAs were observed in *C. trachomatis*-infected host cells in all three treatments. The most dysregulated mRNAs occurred at 20 hpi whiles IFN-γ treatment for 44 hpi showed the least dysregulation of coding genes during *C. trachomatis* infection (Fig. 1g). Taken together, these data suggest that the different stages of *C. trachomatis* infection modulate the expression of distinct sets of host genes.

**Fig. 2:**
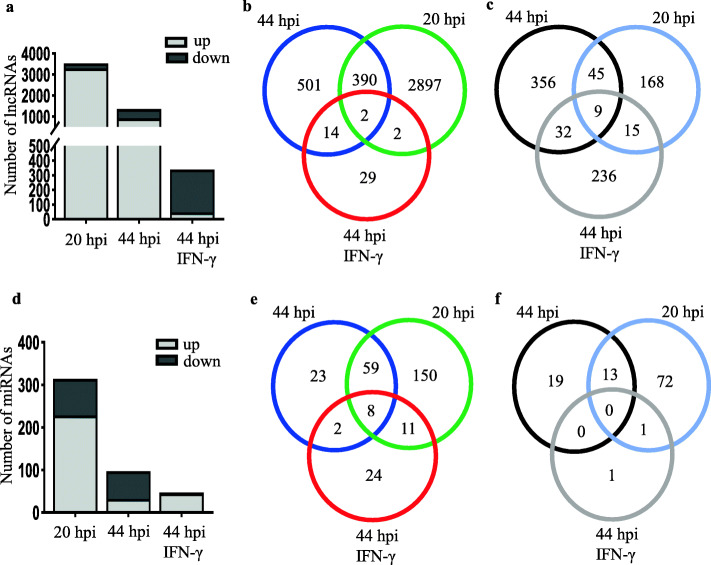
Expression patterns of host cell lncRNAs and miRNAs during *C. trachomatis* development. **a** Differentially expressed lncRNAs in *C. trachomatis*-infected cells at 20 hpi, 44 hpi, and 44 hpi under IFN-γ treatment. **b** Venn diagram of upregulated lncRNAs in *C. trachomatis*-infected host cells. **c** Venn diagram downregulated lncRNAs in *C. trachomatis*-infected host cells. **d** Differentially expressed miRNAs in *C. trachomatis*-infected cells at 20 hpi, 44 hpi, and 44 hpi under IFN-γ treatment. **e** Venn diagram of upregulated miRNAs in *C. trachomatis*-infected host cells. **f** Venn diagram downregulated miRNAs in *C. trachomatis*-infected host cells. The differentially expressed genes are defined as those with fold change ≥2 (upregulated) or ≤ 0.5 (downregulated) between the groups and their respective controls with an adjusted *P*-value of < 0.05. Data are the average from two independent experiments

Next, we analyzed the differentially expressed lncRNAs among infected cells at 20 hpi, 44 hpi, and 44 hpi with IFN-γ treatment. At 20 hpi, we identified 3528 differentially expressed lncRNAs comprising of 3291 upregulated lncRNAs (93.3%) and 237 downregulated lncRNAs (6.7%) (Fig. 2a-c, Supplementary Fig. 2a). At 44 hpi without IFN-γ treatment, 1349 differentially expressed lncRNAs comprising of 907 upregulated lncRNAs (67.2%) and 442 downregulated lncRNAs (32.8%) (Fig. 2a-c, Supplementary Fig. 2b). For 44 hpi IFN-γ treated cells, 339 differentially expressed lncRNAs (47 upregulated and 292 downregulated) were identified as compared to the controls (Fig. 2a-c, Supplementary Fig. 2c). Surprisingly, almost 86% of the overall differentially expressed lncRNAs at 44 hpi with IFN-γ treatment were downregulated as compared to only 7% at 20 hpi and 33% at 44 hpi (Fig. 2c). Among the three stages of *C. trachomatis* infection, 20 hpi and 44 hpi shared 390 upregulated lncRNAs as compared to 14 lncRNAs shared by host cells at 44 hpi without IFN-γ and 44 hpi with IFN-γ treatment. Host cells at 20 hpi and IFN-γ treatment at 44 hpi (Fig. 2b) shared two lncRNAs. For lncRNAs shared by all three treatments, we observed only 2 upregulated and 9 downregulated commonly shared lncRNAs (Fig. 2b-c). Similar to the differentially expressed mRNAs, host cells at 20 hpi showed more differentially upregulated and less downregulated lncRNAs as compared to the other two treatments. Interestingly, the majority of lncRNAs in IFN-γ-treated cells at 44 hpi were downregulated (Fig. 2c) as compared to the host cells at 20 hpi and 44 hpi without IFN-γ treatment in which most of the differentially expressed genes were upregulated indicating reduced transcriptional activation of host genes during persistent infection of *C. trachomatis*.

In *C. trachomatis*-infected host cells at 20 hpi, we observed 314 differentially expressed miRNAs (228 upregulated and 86 downregulated), whiles 124 miRNAs (92 upregulated and 32 downregulated) differentially regulated at 44 hpi (Fig. 2d-f, Supplementary Fig. 3a-b). In the presence of IFN-γ at 44 hpi, we observed 47 differentially expressed miRNAs with only 2 downregulated miRNAs (Fig. 2e-f, Supplementary Fig. 3c). Only 23 miRNAs were uniquely upregulated in the infected cells at 44 hpi as compared to 150 miRNAs at 20 hpi and 24 miRNAs in IFN-γ-treated cells at 44 hpi (Fig. 2e). Furthermore, we observed that only 8 host cell miRNAs were significantly expressed at all developmental stages of *C. trachomatis infection* (Fig. 2e-f). Surprisingly, all three treatments shared no commonly downregulated miRNAs. The highest number of differentially expressed miRNAs was observed at 20 hpi. These data suggest that during *C. trachomatis* infection, the different developmental stages of *C. trachomatis infection* modulate the transcriptional regulation of certain critical non-coding genes that are essential for host cell survival and may play important roles in the pathogenesis of *C. trachomatis*.

### Verification of candidate mRNAs and miRNAs specific for different stages of C. trachomatis infection

**Fig. 3:**
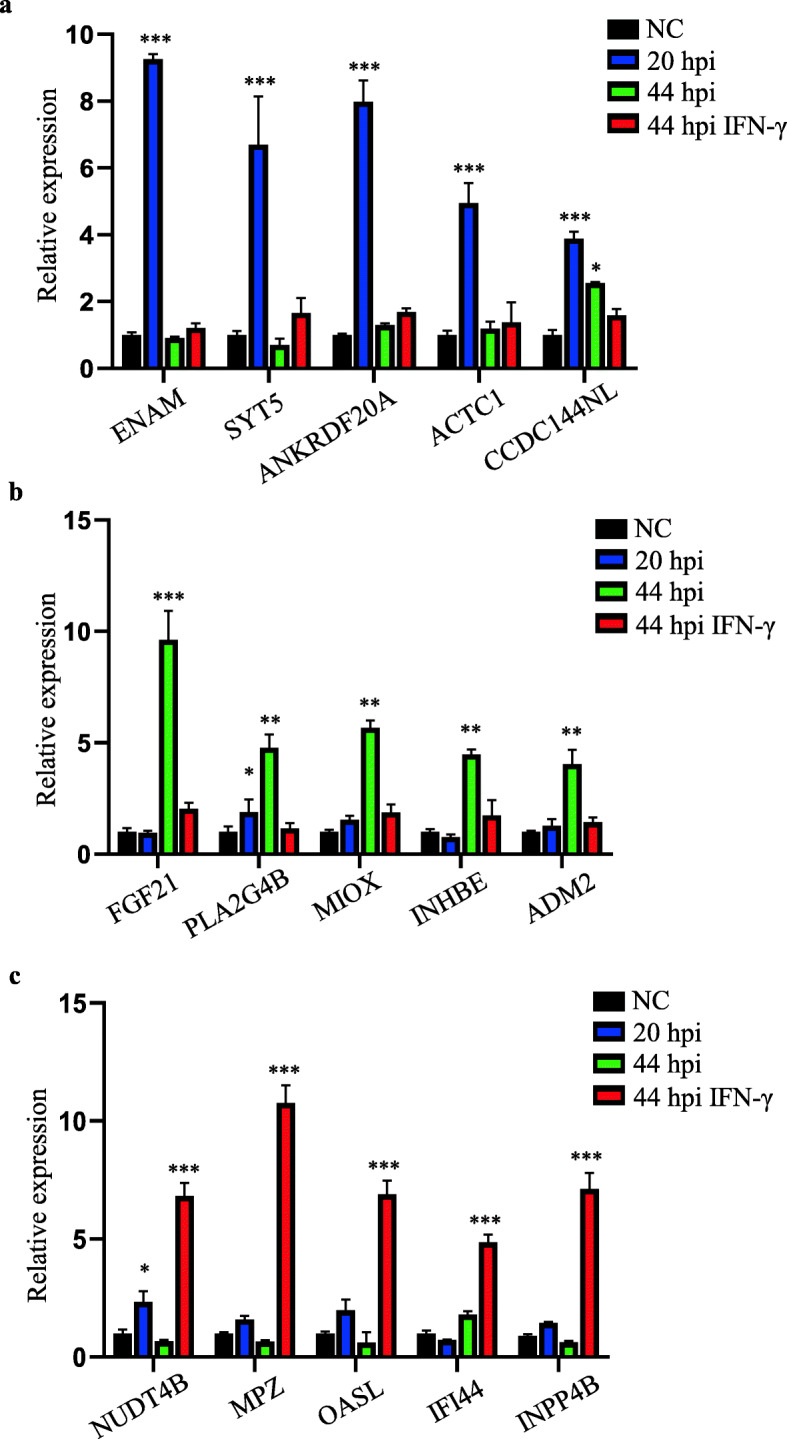
Top 5 most differentially expressed mRNAs in *C. trachomatis-*infected host cells. Relative expression levels of mRNAs at 20 hpi (**a**), 44 hpi without IFN-γ treatment (**b**), and 44 hpi with IFN-γ treatment (**c**). Data are mean ± SEM from three independent experiments with three replicates. Student’s t-test, * *P* < 0.05, ** *P* < 0.01, *** *P* < 0.001

Next, we validated the significantly upregulated mRNAs and miRNAs in *C. trachomatis*-infected host cells at specific stages of infection. Similar to the results obtained from our RNA-seq data, the top 5 upregulated mRNAs showed distinct expression levels at 20 hpi, 44 hpi, and 44 hpi under IFN-γ treatment using a quantitative RT-qPCR. The expression levels of ENAM, SYT5, ANKRDF20, ACTC1, and CCDC144NL were predominantly upregulated at 20 hpi, but not at 44 hpi with or without IFN-γ treatment (Fig. 3a). We found that the expression levels of FGF21, PLA2G4B, MIOX, INHBE, and ADM2 significantly increased at 44 hpi but not at 20 hpi or 44 hpi with IFN-γ treatment (Fig. 3b). Similarly, after IFN-γ treatment for 44 hpi, all the top 5 upregulated host genes (NUDT4B, MPZ, OASL, IFI44, and INP P4B) showed significant expression levels exclusively in the IFN-γ treated host cells as compared to host cells harboring *C. trachomatis* at 20 hpi or 44 hpi without IFN-γ treatment (Fig. 3c).

**Fig. 4:**
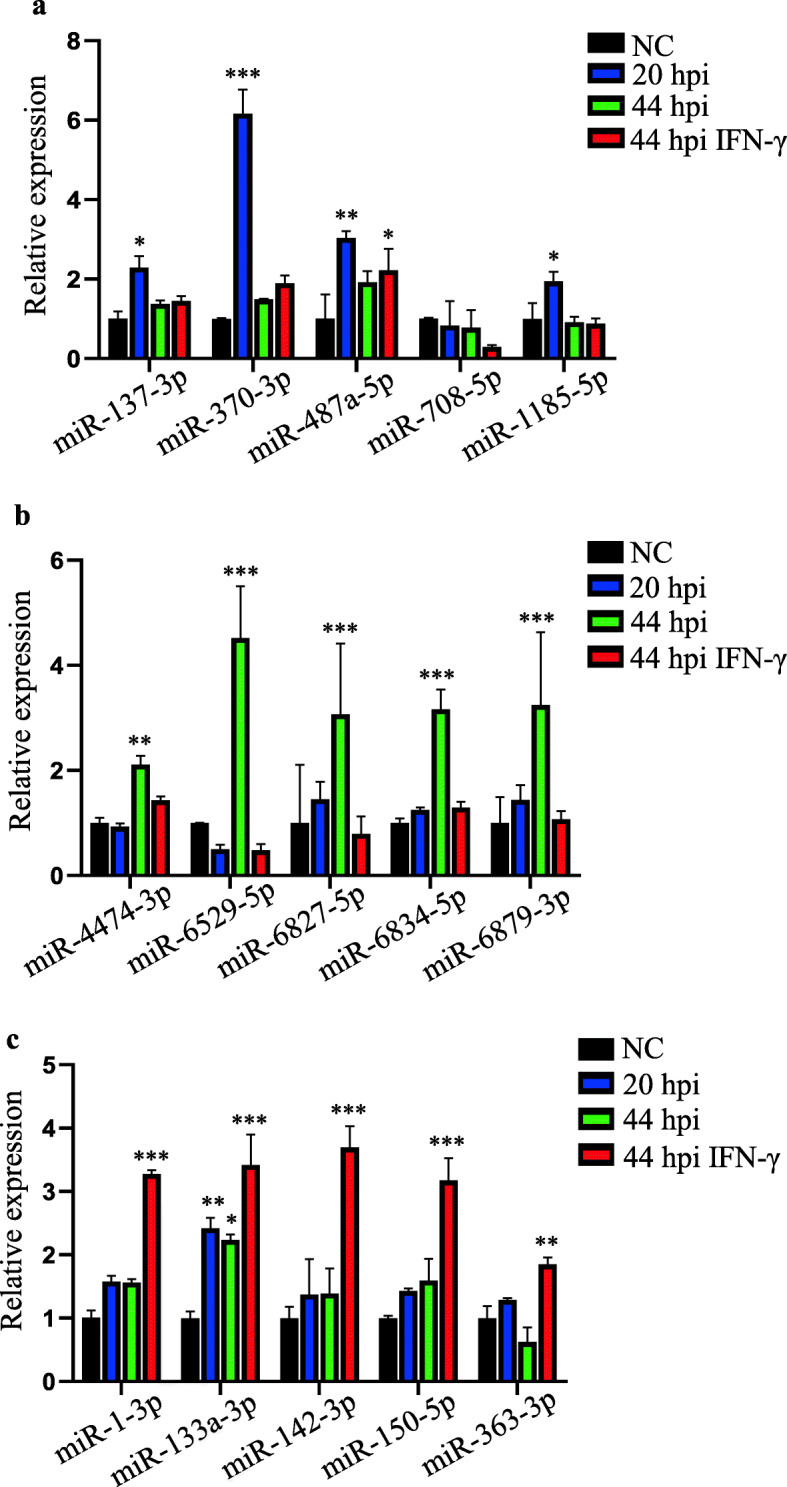
Expression levels of host cell miRNAs during *C. trachomatis*-infection. RT-qPCR analysis of the relative expression levels of the top 5 miRNAs at 20 hpi (**a**), 44 hpi without IFN-γ treatment (**b**), and 44 hpi with IFN-γ treatment (**c**). Data are mean ± SEM from three independent experiments with three replicates. Student’s t-test, * *P* < 0.05, ** *P* < 0.01, *** *P* < 0.001

Furthermore, the top 5 miRNAs identified from the miR-seq data were also validated. At 20 hpi, miRNAs such as miR-137-3p, miR-370-3p, miR-487a-5p, miR-708-5p, and miR-1185-5p were significantly upregulated (Fig. 4a) while miR-4474-3p, miR-6529-5p, miR-6827-5p, miR-6834-5p, and miR-6879-3p were dramatically upregulated in *C. trachomatis*-infected host cells at 44 hpi (Fig. 4b). Among the miRNAs expressed only in the IFN-γ-treated cells at 44 hpi, miR-1-3p, miR-142-3p, miR-150-5p, and miR-363-3p were significantly expressed only at 44 hpi in the presence of IFN-γ. miR-133a-3p showed upregulation at all 3 stages of *C. trachomatis* infection (Fig. 4c). Our results confirmed the hypothesis that *C. trachomatis* infection modulate specific gene expressions at the different stages of *C. trachomatis* infection in host cells.

### miR-193b as a potential serum biomarker of *C. trachomatis* infection

**Fig. 5:**
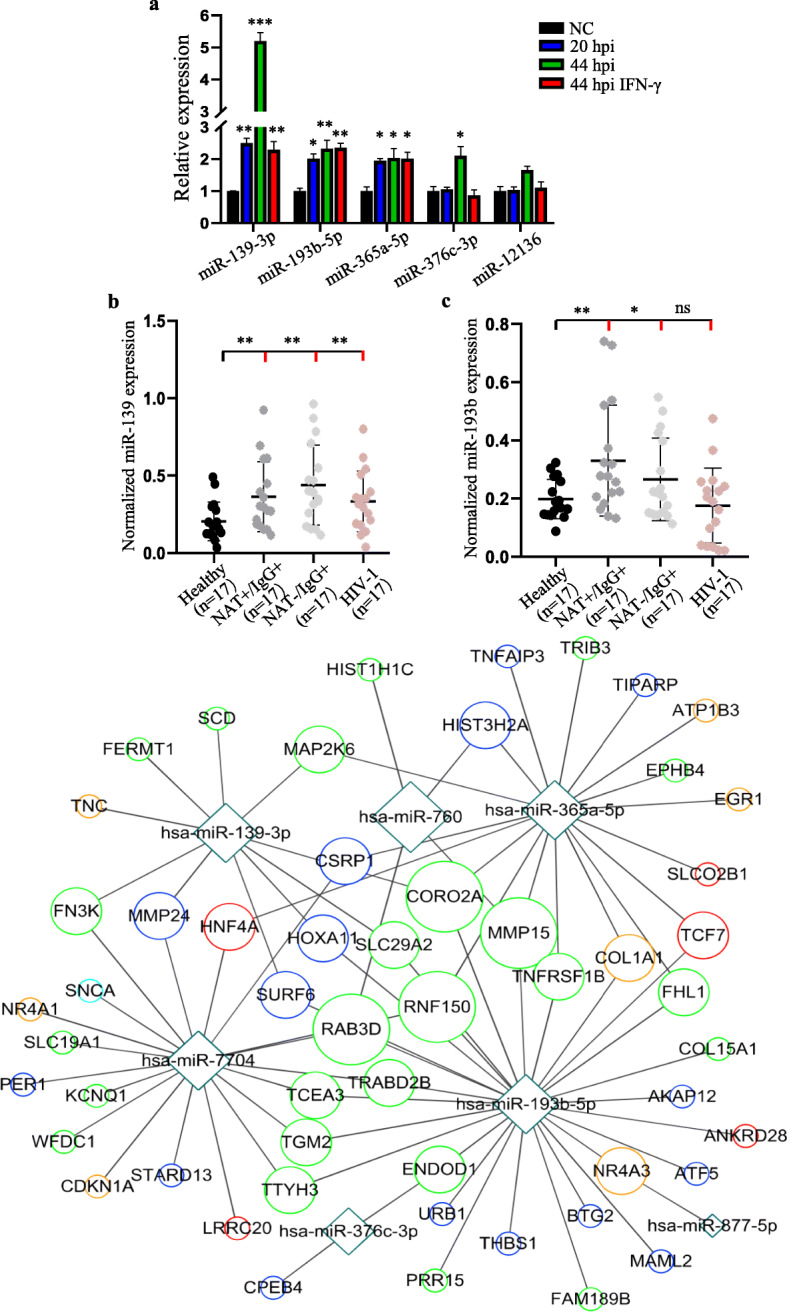
Analysis of commonly expressed miRNAs post-*C. trachomatis* infection*.* **a** Relative expression levels of the top 5 miRNAs that were commonly expressed at all stages of *C. trachomatis* infection. Normalized expression levels of miR-139-3p (**b**) and miR-193b-5p (**c**) in serum samples from *C. trachomatis* patients, HIV-1 patients, and healthy controls. NAT+/Ab+ indicates positive for nucleic acid and *C. trachomatis* antibody. NAT−/Ab+ indicates negative for nucleic acid, but positive for *C. trachomatis* antibody. All serum samples were recovered from − 80 °C after almost a year of storage. Data are mean ± SEM of three replicates for each serum sample (*n* = 17). Relative expressions were calculated using the delta-delta Ct method after normalization with the expression levels of GAPDH in each sample. Student’s t-test, * *P* < 0.05, ** *P* < 0.01, *** *P* < 0.001 by comparing healthy samples (black) with *C. trachomatis* or HIV-1-infected serum samples (red). Images (**a**, **b**, **c**) were created and analyzed with GraphPad Prism 8 (https://www.graphpad.com/scientific-software/prism/). **d** mRNA-miRNA interaction network between the top 5 commonly upregulated miRNAs 20 hpi, 44, and 44 hpi with IFN-γ treatment and their potential targets. The potential miRNA target genes were predicted with TargetScanHuman7.2 software (http://www.targetscan.org/vert_72/). The network was drawn with Cytoscape version 3.0.1 (http://www.cytoscape.org/). Circles indicate the target host genes whereas the squares indicate the miRNAs. The size of the circle represents the degree of the node adjusted according to the number of targeted miRNAs. The color of the circles represents the stage of *C. trachomatis* infection: green, 20 hpi; blue, 44 hpi; red, 44 hpi with IFN-γ treatment; orange, both 20 hpi and 44 hpi with IFN-γ; light blue, 44 hpi with or without IFN-γ

We also tested 5 out of the 8 commonly upregulated miRNAs at 20 hpi, 44 hpi with or without IFN-γ treatment (Fig. 2e), and found that 3 miRNAs (miR-139-3p, miR-193b-5p, and miR-365a-5p) were significantly expressed in *C. trachomatis*-infected host cells (Fig. 5a). Interestingly, the expression levels of miR-139-3p and miR-193b-5p were significantly higher in the serum samples of *C. trachomatis*-infected patients as compared to the sera from healthy blood donors. To determine whether the two miRNAs could differentiate between active and past *C. trachomatis* infections, we tested serum samples from patients who were previously diagnosed by nucleic acid test (NAT) or antibody test for *C. trachomatis*. Both miR-139-3p and miR-193b-5p showed increased expression levels regardless of active *C. trachomatis* infection (NAT+/Ab+) or past infection (NAT−/Ab+) (Fig. 5b-c).

Furthermore, we asked whether these miRNAs are specifically expressed only during *C. trachomatis* infection. Hence, we measured miR-139-3p and miR-193b-5p expression levels in serum samples of previously diagnosed HIV-1-infected patients. We found that miR-139-3p was significantly expressed in HIV-1-infected serum samples whereas miR-193b-5p showed no significant expression in HIV-1-infected serum samples as compared to the serum samples from healthy individuals (Fig. 5b-c). Taken together, these results suggest the involvement of *C. trachomatis* infection in the perturbation of the host genomes leading to the expression of specific miRNAs that may play important roles in maintaining pathogen infection and development. Also, the detection of circulating miRNAs that are specifically expressed in the sera during *C. trachomatis* infection, such as miR-193b-5p, may find potential application in the clinical diagnosis of *C. trachomatis* infection.

To identify the potential target genes of the commonly upregulated host cell miRNAs during *C. trachomatis* infection, we performed competing endogenous RNAs network analysis of significantly upregulated miRNAs at 20 hpi and also at 44 hpi with without IFN-γ treatment and their corresponding downregulated mRNAs. The potential miRNA target genes were predicted with TargetScanHuman7.2 software. Among the commonly upregulated miRNAs at 20 hpi and at 44 hpi with without IFN-γ treatment, miR-193b-5p showed the highest degree of interaction with downregulated mRNAs at 20 hpi (Fig. 5d).

### Identification of stage-specific pathways in host cells

**Fig. 6:**
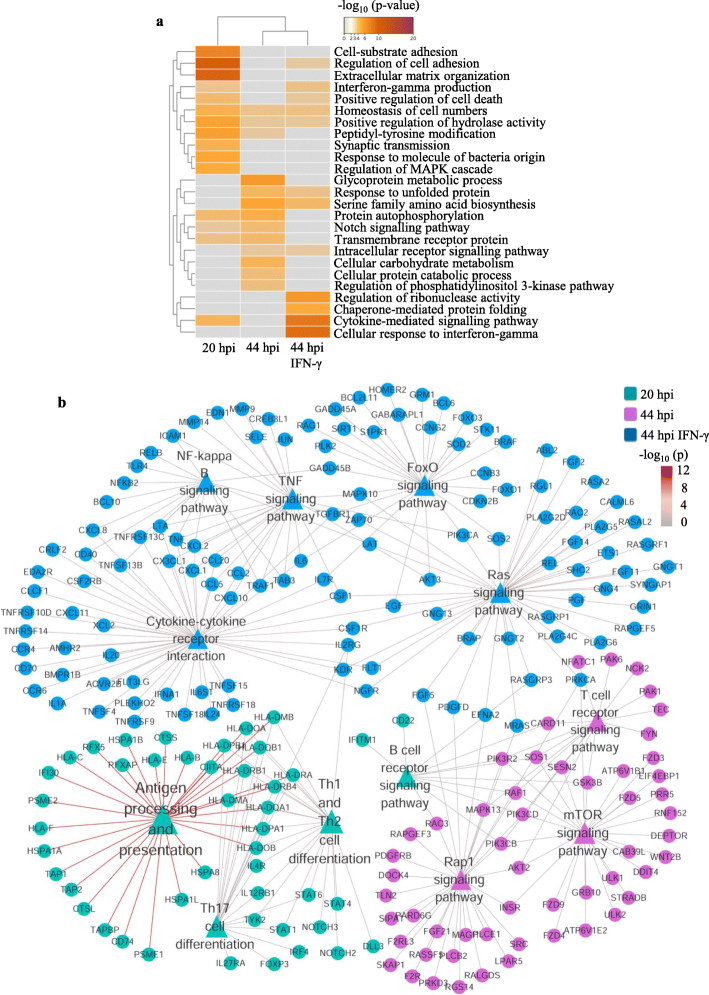
Gene ontology (**a**) and KEGG (**b**) pathways for differentially upregulated host cell mRNAs during *C. trachomatis* infection. Data represent the top 25 biological processes at 20 hpi, 44 hpi, and 44 hpi with IFN-γ. The color of the connecting line is representative of the *p*-value. Images were created with FunRich 3.1.3 (http://funrich.org/)

We then performed gene ontology (GO) annotation and Kyoto Encyclopedia of Genes and Genomes (KEGG) pathway analysis of the differentially upregulated genes during *C. trachomatis* infection. Interestingly, we observed that the differentially upregulated genes at various stages of *C. trachomatis* infection regulate distinct signaling pathways in the host cells (Fig. 6a). Comparison between the biological processes of the host genes at the various stages of *C. trachomatis* infection showed that the host cells at 20 hpi showed the most significantly regulated biological processes such as the regulation of cell adhesion, extracellular matrix organization, and cell-substrate adhesion. Differentially upregulated genes at 44 hpi with IFN-γ treatment were enriched for cytokine-mediated signaling pathways, chaperone-mediated protein folding, and cellular response to IFN-γ. At 44 hpi, host cells exhibited the least regulation of biological process and function (Fig. 6a). Detailed analysis of these pathways revealed that at 44 hpi, the differentially upregulated genes were enriched for the T cell signaling, mTOR signaling, and Rap1 signaling pathways. However, at 20 hpi, most differentially upregulated host genes were essentially involved in antigen processing and presentation. Under IFN-γ treatment at 44 hpi, the host genes were mainly enriched for cytokine-cytokine receptor interaction, FoxO signaling, and Ras signaling pathways (Fig. 6b). Taken together, our analyses showed that different stages of *C. trachomatis* infection modulate distinct host cell signaling pathways and may play critical roles in modulating *C. trachomatis* infection and the survival of the host cells.

## Discussion

With the increasing incidence of *C. trachomatis* infection worldwide, many countries have now considered it a major public health concern. *C. trachomatis* infection results in innate and adaptive immune responses that enable the host cells to attack, destroy, and clear the bacteria [6]. *C. trachomatis* must undergo its biphasic developmental cycle involving an infectious EB and a replicative form RB during the infection of the host cells [5, 6]. Under unfavorable conditions, such as the presence of antibiotics or IFN-γ treatment, *C. trachomatis* may enter the persistent ARB form to enable it to survive. These developmental processes require the synergistic interaction between the host and bacteria genomes [21, 22]. Hence, different stages of *C. trachomatis* infection may require the transcription and post-transcriptional regulation of specific sets of host genes.

RNA sequencing data from host cells infected with *C. trachomatis* indicate that *C. trachomatis* infection modulates dynamic gene expression shift across the genome of the host cells at specific time points during the development cycle of *C. trachomatis*. *C. trachomatis*-infected host cells at 20 hpi showed the most significant host gene dysregulation as compared to 44 hpi with or without IFN-γ treatment (Fig. 1, Fig. 2). Among the dysregulated host genes, we observed 2014, 1388, and 213 mRNAs that were exclusively upregulated 20 hpi, 44 hpi without IFN-γ, and 44 hpi with IFN-γ treatment, respectively, and only 29 commonly regulated genes in all three treatments (Fig. 1). A similar trend was observed for differentially expressed lncRNAs and miRNAs of the host cells at 20 hpi, 44 hpi without IFN-γ, and 44 hpi with IFN-γ treatment suggesting that different stages of *C. trachomatis* infection result in the modulation of a specific set of genes. Generally, a large proportion of mRNAs and lncRNAs were upregulated at 20 hpi whereas *C. trachomatis* infection undergoes active replication into either the infectious or the persistent form. At 44 hpi under IFN-γ treatment, there were very few dysregulated mRNAs, lncRNAs, and miRNAs with the majority of them showing downregulation (Fig. 1d, Fig. 2). These data show that during an active infection, the host cell responds by modifying its transcriptional regulation of genes that are crucial for its survival. However, in the host cells containing the persistent ARB forms of *C. trachomatis* at 44 hpi under IFN-γ treatment, the transcription of a large portion of the host genomes was switched off or at lower levels. These transcriptional switches may probably be due to changes in signaling pathways that are needed for the survival of the pathogen or the clearance of the pathogen at the different stages of infection [23]. Also, the persistent forms of *C. trachomatis* are less active, thereby requiring little nutrient or energy utilization from the host cells.

Circulating miRNAs have been suggested as potential biomarkers of some infectious diseases [24]. Serum miRNAs are relatively stable at variable pH conditions and are highly resistant to enzymatic degradation [25, 26]. These properties make serum miRNAs suitable biomarkers for diagnosis. miR-193b-5p, which was significantly expressed at 20 hpi, and 44 hpi with or without IFN-γ treatment, showed increased expression in the serum of *C. trachomatis*-infected patients only. This observation suggests that miR-193b may serve as a potential serum biomarker for *C. trachomatis* infection (Fig. 5b-c). However, this may require further validation and analysis among a large population of *C. trachomatis*-infected serum samples in future investigations.

Generally, mRNA-miRNA interactions play crucial roles in the modulation of various metabolic pathways in the cells. The importance of several lncRNAs and miRNAs in transcriptional and post-transcriptional regulation of host gene expression has previously been demonstrated [27–29]. Only 8 miRNAs were often upregulated at 20 hpi and 44 hpi with or without IFN-γ treatment (Fig. 2e). However, most miRNAs were upregulated at 20 hpi. Our results are consistent with the fact that active replication of *C. trachomatis* occurs within the first 20–24 h of *C. trachomatis* infection [6].

Further analysis of the differentially upregulated genes showed enrichment of distinct signaling pathways at the specific stages of *C. trachomatis* infection. There was little overlap in the pathways regulated by differentially upregulated host genes at 20 hpi, 44 hpi, and 44 hpi with IFN-γ treatment (Fig. 6). Host cells at 20 hpi revealed the dysregulation of biological processes such as cell adhesion, extracellular matrix organization, and cell-substrate adhesion as previously reported [30]. The most significantly regulated pathways in cells at 44 hpi under IFN-γ treatment were cytokine-mediated signaling pathway and chaperone-mediated protein folding (Fig. 6). The upregulation of these pathways in host cells infected with the persistent forms of *C. trachomatis* may serve as immune surveillance or manipulation of the host immune system to allow the pathogen to escape [31].

Previous reports have shown that *C. trachomatis* infection leads to the activation of the ERK MAPK, PI3K, and RAS signaling pathways, which are major regulatory pathways in the nutrient cycle in bacteria infections [32, 33]. Also, chlamydial infection interferes with the immune response of the host cells to viral infections and the subsequent fate of the cells [33]. Detailed analysis revealed that the differentially upregulated host genes at 20 hpi were enriched for antigen processing and presentation while at 44 hpi, host genes were enriched for T cell receptor signaling, mTOR signaling, and Rap1 signaling pathways. In the presence of IFN-γ, the most significantly activated signaling pathways in host cells were cytokine-cytokine receptor interaction, FoxO signaling, and Ras signaling pathways (Fig. 6b), as previously reported [33]. Thus, at the various stages of Chlamydia development, there is the modulation of different components of essential host cell signaling pathways that support the survival of the pathogen [33]. Hence, a transcriptional manipulation of completely different sets of host genes and pathways at the various stages of *C. trachomatis* infection may be important in the survival of the host cells as the complexity and content of the inclusion body changes [23]. The activation of these essential signaling pathways in the host cells at the various stages of *C. trachomatis* infection suggests that *C. trachomatis* induces a combination of pathways that are responsible for the survival of the host cells. The specific role of the differentially regulated host genes at each developmental stage of *C. trachomatis*-infection remains to be investigated.

## Conclusions

Our current study demonstrated that *C. trachomatis* infection of HeLa cells modulates the expression levels of mRNAs, lncRNAs, and miRNAs, which shows specificity for different stages of *C. trachomatis* infection and may influence the host cell response to infection. The dysregulation of these stage-specific host genes results in the differential regulation of important signaling pathways that are necessary for the survival of the pathogen. Serum expression of miR-193b could serve as a potential serum biomarker in the diagnosis of *C. trachomatis* infection for improved management and control of the disease. RNA-seq and miR-seq data obtained from this study could also serve as an essential resource for future investigations into the molecular mechanisms at specific developmental stages of *C. trachomatis*.

## Methods

### Cells, bacteria, and clinical samples

HeLa229 cells were purchased from American Type Culture Collection (Manassas, VA, USA) and cultured in Dulbecco’s modified Eagle’s medium (DMEM) supplemented with 10% heat-inactivated fetal bovine serum (Sigma-Aldrich, USA) at 37 °C with 5% CO_2_. *C. trachomatis* serovar E was a kind gift from the Clinical Microbiology Laboratory of the Chenzhou No. 1 People’s Hospital (Chenzhou, China) and was routinely preserved in our laboratory.

### Infection of HeLa cells

HeLa cells were infected with *C. trachomatis* as previously described [34] with little modification. Briefly, 1 × 10^5^ HeLa cells in 6-well plates were cultured overnight. Cells were first treated with DEAE-Dextran at 30 mg/L before infection with *C. trachomatis* at a multiplicity of infection (MOI) of 1 by centrifugation onto the host cells at 1000 rpm for 1 h. The MOI was calculated using the inclusion-forming unit (IFU) with MOI = IFU/number of cells. EBs were purified and preserved in a sucrose-phosphate-glutamate buffer before storage at − 80 °C. For RNA extraction, *C. trachomatis*-infected host cells at the various stages of *C. trachomatis* infection were collected as follows: 20 hpi, 44 hpi, and 44 hpi with IFN-ɣ (50 units) treatment immediately after infection. Uninfected HeLa cells were used as respective negative controls at 20 and 44 hpi, and IFN-ɣ-treated cells were used as negative controls for 44 hpi IFN-ɣ-treated *C. trachomatis*-infected cells.

### Immunofluorescence assay

Cells were washed twice with PBS followed by incubation with 4% formaldehyde at room temperature for 30 mins. After a single wash, cells were then incubated with 0.1% Triton X-100 at room temperature for 10 mins and washing three times with PBS. Wells were blocked with 4% bovine serum albumin (BSA) for 1 h followed by incubation with 2 μg/ml anti-MOMP antibody (Abcam, Cambridge, UK) and 1:1000 dilution of DAPI (Sigma, USA) at 37 °C for 1 h. Cells were washed thrice with PBS and then incubated with 2μg/ml CoraLite 594 fluorescence anti-mouse IgG (Proteintech, USA) for 30 mins at 37 °C. Cells were observed after washing three times with PBS. Images were obtained with.

### RNA extraction

Total RNAs from *C. trachomatis*-infected cells were extracted using TRIzol L/S (Invitrogen, USA) according to the manufacturer’s instructions with few modifications, as described previously [29]. Serum miRNAs were extracted using the miRNeasy Serum/Plasma Kit (Qiagen Inc., Germany) according to the manufacturer’s instruction. Briefly, frozen serum samples were thawed on ice and 200 μl of each serum sample was mixed thoroughly with 1 ml QIAzol lysis reagent. Chloroform (200 μl) was added and incubated at room temperature for 5 min. Tubes were centrifuged for 15 min at 12,000×g at 4 °C. The aqueous phase was then mixed with 900 ml of 100% ethanol. About 700 μl of the mixture was loaded into the RNeasy minElute spin column in a 2 ml collection tube and followed by centrifugation. The column was then washed with 700 μl of Buffer RWT, 500 μl of Buffer RPE, and 500 μl of 80% ethanol. RNA was eluted with 14 μl RNase-free water.

### RNA sequencing and analysis

For next-generation RNA sequencing, sequencing libraries were constructed as previously described with modifications [35] and sequencing was carried out with the BGISEQ-500 system at the Beijing Genome Institute (Wuhan, China). The quality of the raw sequence reads was assessed using FastQC [36]. Sequenced reads were trimmed for adaptor sequence, and masked for low-complexity or low-quality sequences, then mapped to hg38 whole genome using bowtie v2.2.5 with parameters -q --phred64 --sensitive --dpad 0 --gbar 99,999,999 --mp 1, 1 --np 1 --score-min L,0,-0.1 -I 1 -X 1000 --no-mixed --no-discordant -p 1 -k 200, and RSEM v1.2.12 with parameters --forward-prob 0. Raw data were processed using the Zebra call and CASAVA pipeline softwares. For miRNA-seq, the data were mapped to the hg38 whole genome and the miRNA database in miRBase with bowtie (−v 1). Data normalization and differential analysis were done using the default methods in the DESeq R package [37]. The read counts of samples were normalized for sequencing depth and distortion caused by highly differentially expressed genes. A negative binomial model was used to test the significance of differential expression between the two conditions. A cutoff of False Discovery Rate (FDR) of less than 0.05 and a log fold change > 2.0 was used to select the significant DEGs by computing the *p*-values.

### Quantitative RT-PCR analysis

First-strand cDNA was synthesized with 500 ng of total RNA using TransScript All-in-One First-Strand cDNA Synthesis SuperMix (Transgen Biotech, China). For mRNA validation, primers spanning 70–200 bp of the mRNA sequence were designed and used in the validation. MiRNA specific RT-primers were used for first-strand cDNA synthesis of 500 ng of serum-extracted miRNA followed by the use of universal miRNA reverse primer and miRNA specific forward primers for miRNA validation. For RT-qPCR, the SYBR Green qPCR Master Mix (Accurate Biotechnology, China) with cycling conditions of 95 °C for 5 min followed by 50 cycles of 95 °C for 10 s, 60 °C for 10 s, and 72 °C for 10 s in the LightCycler 96 SW 1.1 real-time PCR system (Roche, USA) was used according to standard procedures. GAPDH was used as control. A list of primers is provided in Supplementary Table 1.

Gene ontology (GO) annotation and Kyoto Encyclopedia of Genes and Genomes (KEGG) pathway enrichment analysis.

GO functional annotation was carried out using the Gorilla web-server [38]. The Enrichr database (http://amp.pharm.mssm.edu/Enrichr/) was utilized KEGG pathway enrichment analysis of upregulated mRNAs [39]. *P*-values were calculated with default parameters.

### Statistical analysis

All RNA-seq data are from two biological replicates. Quantitative data were analyzed using GraphPad Prism from three independent experiments. The miRNA-mRNA network was illustrated with Cytoscape [40].

## Supplementary Information

**Additional file 1: Supplementary Fig. 1** Differentially expressed mRNAs in *C. trachomatis*-infected HeLa cells at 20 hpi **(a)**, 44 hpi **(b)**, and 44 hpi with IFN-γ treatment **(c)**. The cutoff criteria used in the volcano plot were a fold-change of 2 with a *P*-value of <0.05. Log_2_ of average reads from two biological replicates were plotted. The heatmap plot shows the upregulated host gene expression with cutoff value >1 and P-value <0.05.

**Additional file 2: Supplementary Fig. 2** Scatter plot of lncRNAs in cells containing *C. trachomatis* at 20 hpi **(a)**, 44 hpi **(b)**, and 44 hpi with IFN-γ treatment **(c)**. The criteria used were a fold-change of ≥ 2 or ≤ -2, and a P-value of <0.05. Log_2_ of average reads from two biological replicates were plotted.

**Additional file 3: Supplementary Fig. 3** Scatter plot of miRNAs in cells containing *C. trachomatis* at 20 hpi **(a)**, 44 hpi **(b)**, and 44 hpi with IFN-γ treatment **(c)**. The criteria used were a fold-change of ≥2 or ≤-2, and a P-value <0.05. Log_2_ of average reads from two biological replicates were plotted.

**Additional file 4: Supplementary Table 1** List of Primers used in this study.

## Data Availability

RNA-seq/miR-seq data generated in this study have been deposited in NCBI’s Gene Expression Omnibus and are accessible through GEO accession number GSE158814.
