# Supplementary figures and images for: Host cell response and distinct gene expression profiles at different stages of Chlamydia trachomatis infection reveals stage-specific biomarkers of infection
Source: BMC Microbiol. 2021 Jan 4;21:3. doi: 10.1186/s12866-020-02061-6 (PMC7784309; doi:10.1186/s12866-020-02061-6)

## Supplementary Fig. 1

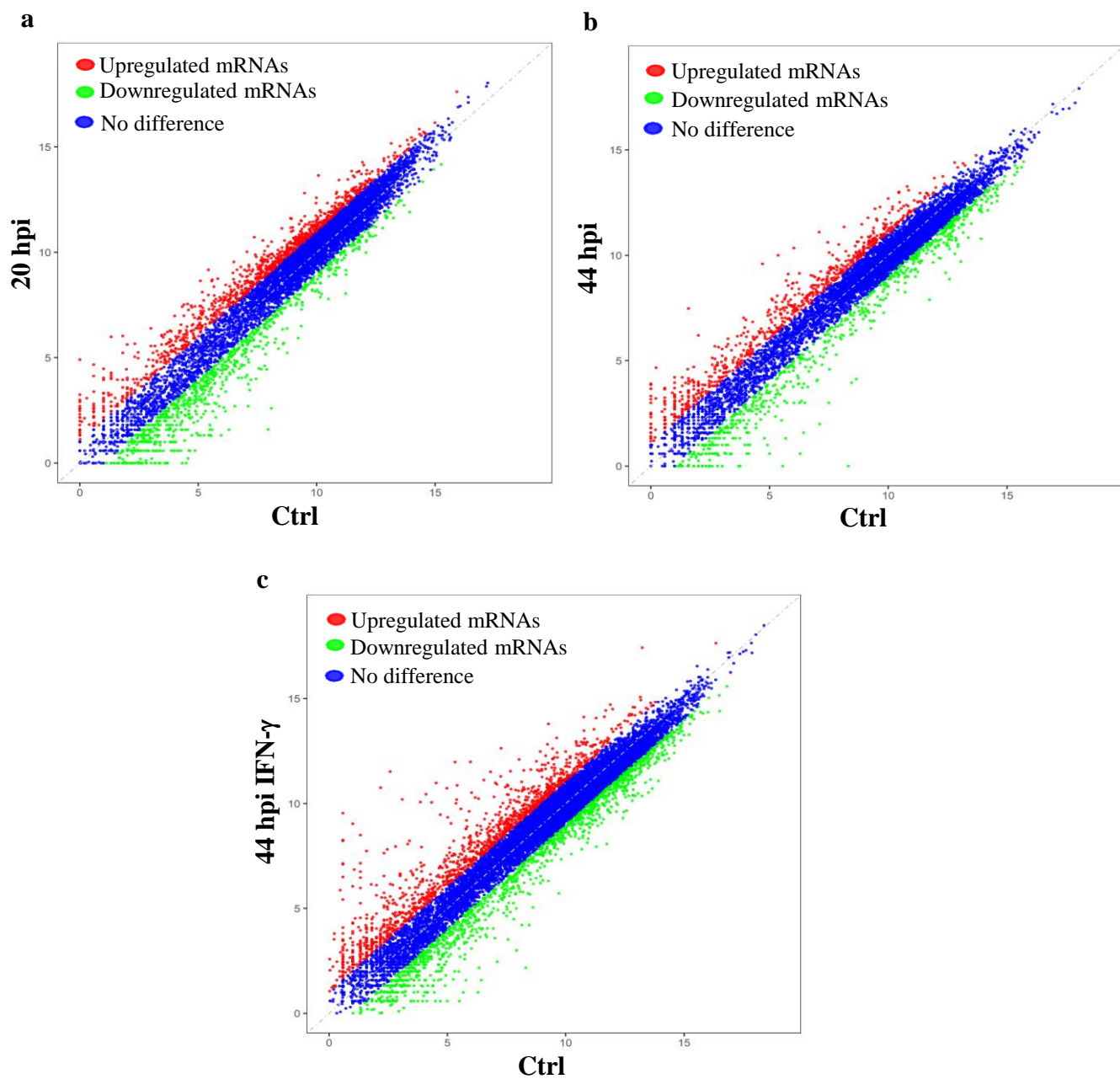

Supplement: Supplementary file 1 — Additional file 1: Supplementary Fig. 1 Differentially expressed mRNAs in C. trachomatis-infected HeLa cells at 20 hpi (a), 44 hpi (b), and 44 hpi with IFN-γ treatment (c). The cutoff criteria used in the volcano plot were a fold-change of 2 with a P-value of <0.05. Log2 of average reads from two biological replicates were plotted. The heatmap plot shows the upregulated host gene expression with cutoff value >1 and P-value <0.05. [file 12866_2020_2061_MOESM1_ESM.pdf]

## Supplementary Fig. 2

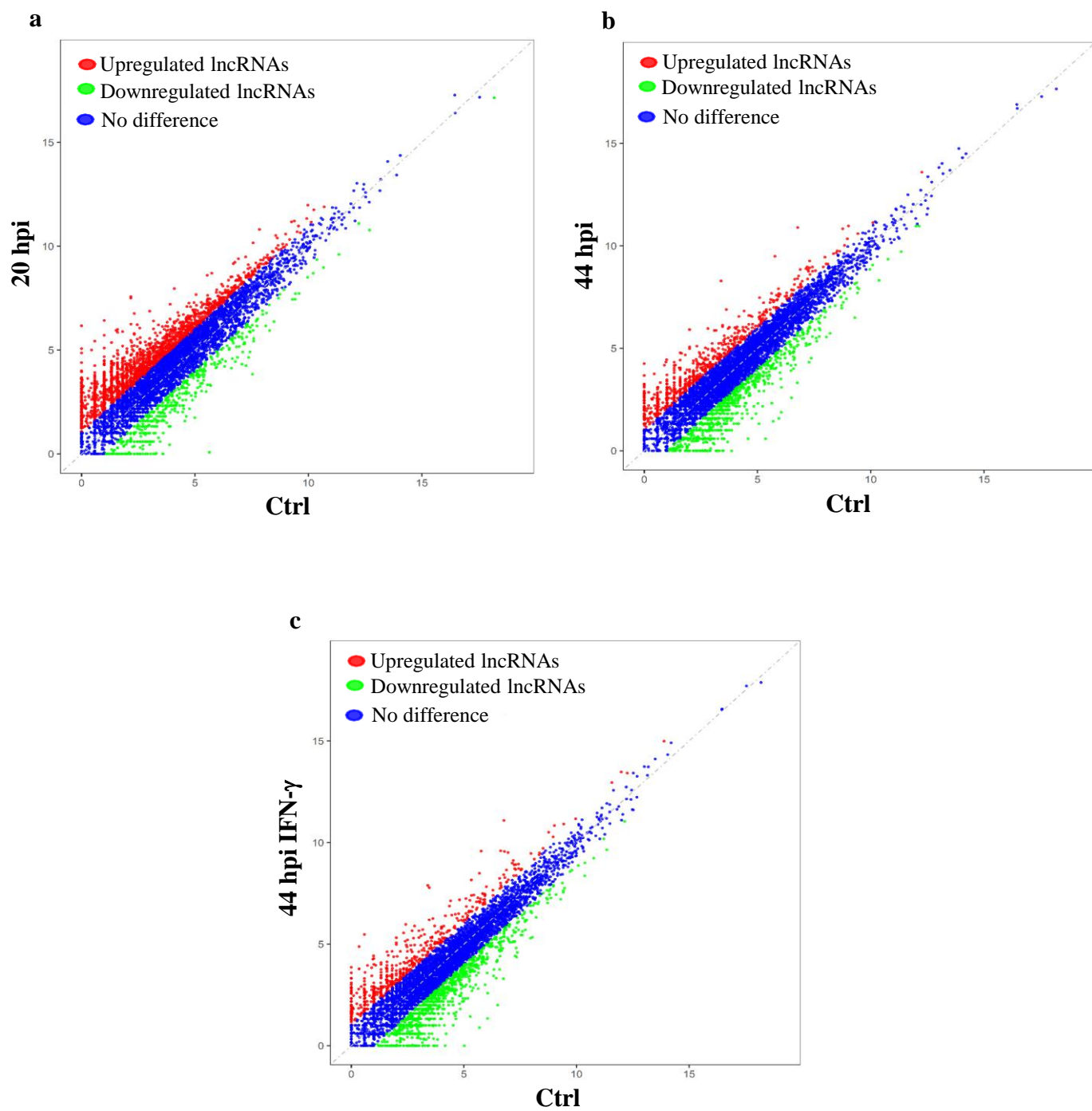

Supplement: Supplementary file 2 — Additional file 2: Supplementary Fig. 2 Scatter plot of lncRNAs in cells containing C. trachomatis at 20 hpi (a), 44 hpi (b), and 44 hpi with IFN-γ treatment (c). The criteria used were a fold-change of ≥ 2 or ≤ -2, and a P-value of <0.05. Log2 of average reads from two biological replicates were plotted. [file 12866_2020_2061_MOESM2_ESM.pdf]

# Supplementary Fig. 3

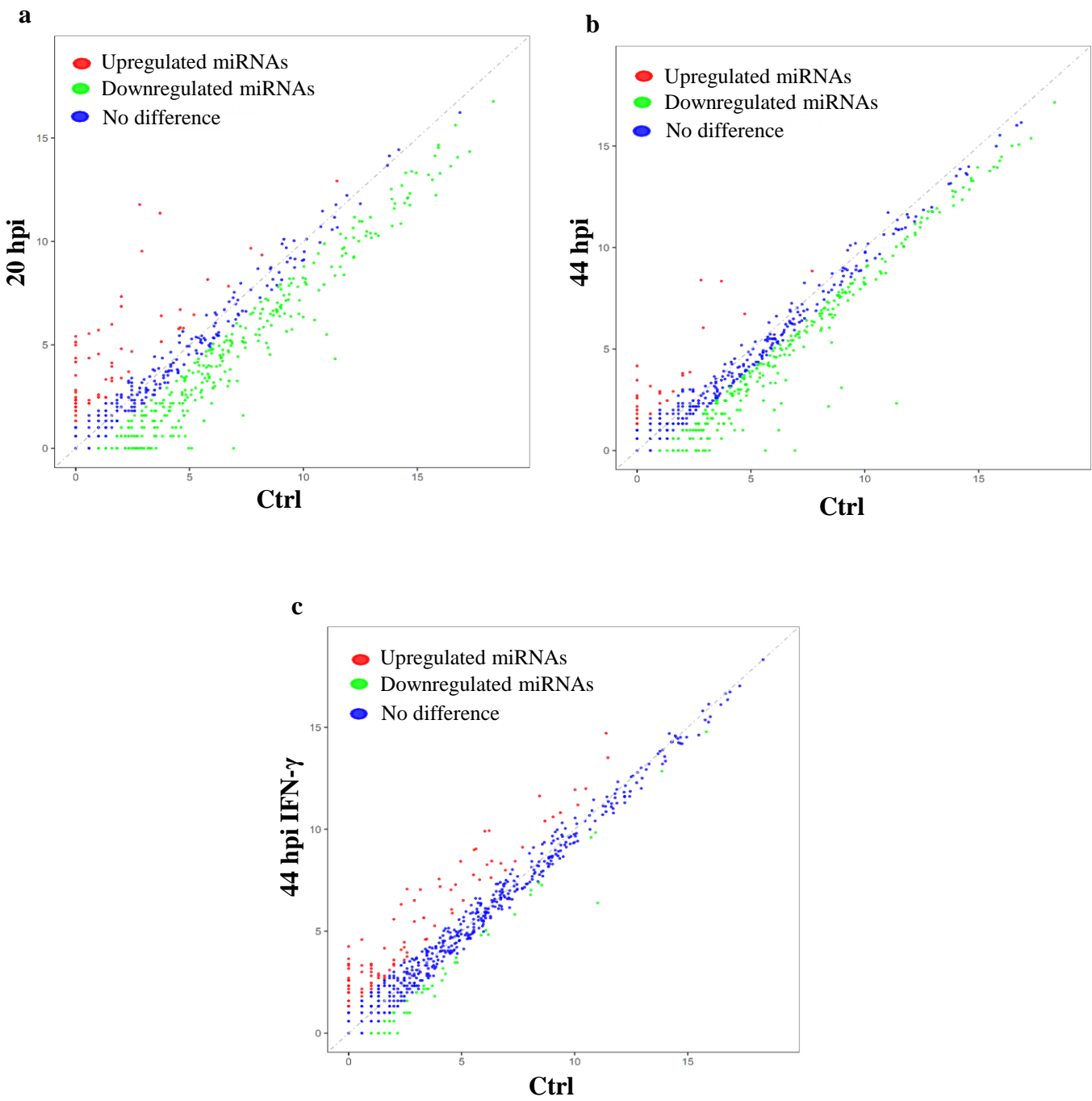

Supplement: Supplementary file 3 — Additional file 3: Supplementary Fig. 3 Scatter plot of miRNAs in cells containing C. trachomatis at 20 hpi (a), 44 hpi (b), and 44 hpi with IFN-γ treatment (c). The criteria used were a fold-change of ≥2 or ≤-2, and a P-value <0.05. Log2 of average reads from two biological replicates were plotted. [file 12866_2020_2061_MOESM3_ESM.pdf]
